# Predictors of Intention to Quit Waterpipe Smoking: A Survey of Arab Americans in Houston, Texas

**DOI:** 10.1155/2015/575479

**Published:** 2015-03-04

**Authors:** Liqa Athamneh, Sujit S. Sansgiry, E. James Essien, Susan Abughosh

**Affiliations:** ^1^Department of Pharmaceutical Health Outcomes and Policy, Texas Medical Center, University of Houston, 1441 Moursund Street, Houston, TX 77030, USA; ^2^The University of Texas School of Public Health, 7000 Fannin Street, Houston, TX 77030, USA

## Abstract

Waterpipe smoking has been described as “the second global tobacco epidemic since the cigarette.” Both Middle Eastern ethnicity and having a friend of Middle Eastern ethnicity have been reported as significant predictors of waterpipe smoking. Addressing waterpipe smoking in this ethnic minority is essential to controlling this growing epidemic in the US. We investigated the predictors of an intention to quit waterpipe smoking by surveying 340 Arab American adults in the Houston area. Primary analyses were conducted using stepwise logistic regression. Only 27% of participants reported having an intention to quit waterpipe smoking. Intention to quit waterpipe smoking was significantly higher with history of cigar use, a prior attempt to quit, and not smoking when seriously ill and significantly lower with increasing age, medium cultural acceptability of using waterpipe among family, high cultural acceptability of using waterpipe among friends, longer duration of smoking sessions, and perceiving waterpipe smoking as less harmful than cigarettes. Educational programs that target Arab Americans in general, and specifically older adults, those who smoke waterpipe for more than 60 minutes, those whose family and friends approve waterpipe smoking, and those with no former attempts to quit, may be necessary to increase the intention to quit waterpipe smoking.

## 1. Introduction

The term waterpipe refers to any type of instruments that involve passing tobacco smoke through water before inhalation [1]. Using waterpipe to smoke tobacco is a centuries-old habit that has been practiced in Asian and African regions but is usually linked with the eastern Mediterranean area [2].

Waterpipe smoking is currently the object of renewed attention, as its use has been gaining popularity lately among young people in western countries, such as the US, Europe, and Brazil [3]. During the past decade, it has been described as the “second global tobacco epidemic since the cigarette” [4].

In the US, reports show that waterpipe smoking is rising [5] and becoming the most common type of tobacco smoked by US adults after cigarettes [6, 7]. Waterpipe smoking has been reported in more than 33 states, with most reports deriving from cities with a large university [8, 9]. In a study targeting 6,594 students of the state of Arizona (grades 6 through 12) about 4% of the students were current waterpipe smokers (7.3% of 12th grade students) and 6% of students had previously used a waterpipe [10]. A survey of 411 Johns Hopkins University freshmen found that about 15% of students reported waterpipe tobacco smoking in the past 30 days [11]. Another survey of 647 students from graduate and undergraduate programs at a large urban university in the US found about 10% of participants reported current, and 41% had previous waterpipe smoking [12]. Another study of 2,204 students at the University of Houston reported that 12.5% of the sample participants had smoked with a waterpipe within the last month [13].

Waterpipe smokers are at the same risk of smoking diseases such as lung cancer, oral cancer, cancer of the esophagus, stomach cancer, decreased fertility, and reduced lung function as cigarette smokers [14, 15].

Being of Middle Eastern ethnicity or having a friend of Middle Eastern ethnicity has been reported as significant predictors of waterpipe smoking among US students [16]. Consequently, addressing waterpipe smoking in this ethnic minority is essential in controlling this growing epidemic in the general US population.

Arab Americans are one of the rapidly growing minority groups in the USA for which there seems to be little or no smoking behavior data. The US Census Bureau categorizes Arab Americans as people with descents originating from Arab-speaking areas of the world and considers them as one of the fastest growing populations in the USA [17]. There are approximately 1.5 million Americans (0.5 percent of the total population) who have an Arab origin [17]. They usually derive from common linguistic, cultural, and political heritages.

Smoking in Arab countries is an acceptable cultural and social behavior; it is a sign of hospitality to offer someone a cigarette, and it is a normal behavior to smoke inside the home [18]. In a study that was conducted on Arab American adolescents, the participants described their use of waterpipe in the home as an acceptable social activity by their parents [19].

The stage-based model of behavioral change was indicated to be effective for cigarette smoking cessation [20, 21]. According to the model, the modification of addictive behaviors includes progression through five stages: precontemplation stage with no intent to quit, contemplation stage with starting an intention to quit, preparation for the behavioral change, action and change, and finally maintaining the change [22].

As forming an intention to quit is the first step of quitting, identifying predictors of an intention to quit is integral in designing effective intervention strategies to aid waterpipe smokers in this subpopulation in quitting and prevent further diffusion into the US society.

To date, no studies have investigated the predictors of an intention to quit waterpipe smoking among the Arab-American population. Our study's objective is to investigate the predictors of intention to quit waterpipe smoking in the next 12 months among a sample of Arab Americans who smoke tobacco using a waterpipe in the Houston area.

## 2. Methodology

### 2.1. Study Design, Setting, and Area

To address the study goals, an observational, survey-based cross-sectional study with a convenience sample of Arab American adults was conducted.

The study was carried out in Houston, Texas, with a population of more than 2,1 millions in 2013 [23]. Houston was chosen as it is one of the ten places in the United States that have the largest Arab population, with about 41,653 people from Arab origins living in Houston in 2013 [23, 24] and also because it was convenient for the investigator who is living in this area. Data were collected by distributing surveys to willing adult participants from Arab origins in the study area.

### 2.2. Study Procedures

Adults visiting Arab congregations or hookah bars (regardless of their skin color or any physical characteristics) were introduced to the study and the target population and asked if they considered themselves to be among this group of people. If they answered yes, they were asked if they were willing to participate in filling out an anonymous survey regarding their waterpipe smoking habits. The basic information about the study, such as the purpose, procedures, confidentiality issues, risks, and benefits to participation both verbally and in written form, was explained by the investigators before filling out the questionnaire. Filling out and returning the questionnaire implied the study participants' consent. When a qualified person refused to participate in filling the questionnaire, the investigator kindly asked for the reason and noted the reason given.

Inclusion criteria for the study were adult aged 18 years or older, from Arab American descent, and smoking a waterpipe in the last 30 days [25]. There was a locked box in the data collection sites to drop off the survey upon completion. As the surveys were anonymous, there were no confidentiality issues.

### 2.3. Sample Size

The minimum effective sample size that is required for the study was determined using G∗POWER 3.1 statistical software package [26]. Considering a multiple logistic regression statistical test at 95% confidence, 80% power, and for a 1.5 odds ratio, the required sample size for this study was estimated to be 307.

### 2.4. Ethical Considerations

The study received approval from the Institutional Review Boards (IRBs) and the Committee for the Protection of Human Subjects (CPHS) at the University of Houston.

### 2.5. Data Collection Instrument

The study used a self-administered survey. The questionnaire consisted of five major sections with “yes/no” and multiple-choice questions. The survey was adopted from previously used and validated questionnaires [11, 13, 27, 28].

The first 6 questions cover the demographics including age, gender, annual income, marital status, and education level. Questions 6–30 were taken from a survey that was originally developed by Smith and coworkers [11, 27] and include questions about tobacco use history, perception of risk, perceived social acceptability of smoking tobacco using a waterpipe, and waterpipe-related practices such as the ownership of waterpipe, frequency and length of smoking sessions, and age and place of first waterpipe use. Items 31–41 were about the addiction behavior and its four subscales: the nicotine dependence, negative reinforcement, psychological craving, and positive reinforcement properties of waterpipe smoking.

### 2.6. Statistical Analysis

Descriptive statistics and chi-square analyses were used to determine the frequencies and associations of sample characteristics with the intention to quit waterpipe smoking. The intention to quit was determined using the question (Do you intend to quit using a waterpipe to smoke tobacco in the next 12 months? Yes or No).

Bivariate analyses of sample characteristic were carried out with the outcome variable (intention to quit waterpipe smoking in the next 12 months versus not), and results were presented as unadjusted odds ratios (OR) with 95% confidence intervals (CI). After assessing colinearity between the independent variables (IVs), all IVs with *P* < 0.2 in the unadjusted analysis were included in the stepwise multivariate logistic regression model to determine predictors of intention to quit waterpipe smoking.

All the statistical analysis was conducted using SAS 9.2 (SAS Institute Inc., Carey, North Carolina) at a significance level of 0.05.

## 3. Results 

A total of 340 participants completed questionnaires. Overall, the prevalence of having an intention to quit waterpipe smoking among this study sample was 27.43% (Table 1).

### 3.1. Response Rate

Data collection was completed between mid-January to mid-March, 2014. Data was collected from adults, visiting hookah bars, public areas, Arab congregations, or mosques in Houston. The total responses collected at the end of the data collection were 340 out of 510 surveys distributed. Thus the overall response rate was 67%, with “lack of time” being the major reason reported (by 99%, *N* = 168) for not participating in the study.

### 3.2. Sociodemographic Data

The distribution of the sociodemographic characteristics of the waterpipe smokers that participated is shown in Table 1. The study sample consisted mainly of males (67%), married (50%), with a mean age (±SD) of 30 ± 8.5 (range 18–65) years. The chi-square test results showed no significant association between the intention to quit waterpipe smoking in the next 12 months and gender, income level, marital status, or education.

### 3.3. Smoking Patterns

The mean age (±SD) of starting to smoke tobacco using a waterpipe was 20 (±6.5) (range 3–55 years old). Ninety-six percent of the participants initiated waterpipe smoking in the company of their family members or friends. Two-thirds of participants own a waterpipe and 66% share it with others. Ninety-one percent reported smoking flavored tobacco in the waterpipe. Sixty percent of participants smoke waterpipe tobacco at least once a week (38% smoke it at least once a day) (Table 1).

### 3.4. Tobacco Use History

Approximately 49% of the participants had smoked cigarettes and 31% had smoked cigars, cigarillos, or little cigars in the last 30 days. By calculating the frequency of those who answered yes to all three questions: did you smoke waterpipe in the last 30 days? Did you smoke cigar, cigarillos, or little cigars in the last 30 days? Did you smoke cigarettes in the last 30 days? 87% of the study sample reported using cigarette, cigar, and waterpipe in the last 30 days to smoke tobacco. The chi-square results listed in Table 1 showed no significant association between the intention to quit waterpipe smoking in the next 12 months and cigarette or cigars use history (Table 1).

### 3.5. Perception of Risk

Thirty-two percent of participants perceived waterpipe smoking as less harmful to one's health compared to cigarette smoking. The chi-square test results indicate that the intention to quit waterpipe smoking in the next 12 months is significantly associated with perception of waterpipe smoking risk compared to cigarettes (*P* = 0.0022) (Table 2).

### 3.6. Perceived Social Acceptability

Few participants reported that their families (14.8%), friends (9.6%), and peers (10.9%) did not approve or accept their use of waterpipe tobacco.

Chi-square analyses indicated a significant association between an intention to quit waterpipe smoking in the next 12 months with the cultural acceptability of waterpipe smoking among family members and friends from the same ethnicity (*P* = 0.0028 and 0.0158, resp.). In the study sample, almost two-thirds of participants believed that waterpipe smoking looks cool or very cool (Table 3).

### 3.7. Waterpipe-Related Practices and Beliefs

Shorter length of smoking session (*P* = 0.0004), higher number of times the participants stopped waterpipe smoking for more than 7 days (*P* = 0.0137), pleasure as not a reason to smoke (*P* = 0.0189), and pleasing others as a reason to smoke (*P* = 0.0264) were significantly associated with an intention to quit waterpipe smoking in the next 12 months (Table 4).

### 3.8. Logistic Regression Results

After controlling for the covariates, the final results for the stepwise multiple logistic regression indicated that the significant predictors associated with a higher intention to quit waterpipe smoking in the next 12 months among Arab Americans are history of cigar use in the last 30 days compared to those who did not use cigars in the last month (OR: 4.38 CI: 1.86–10.31), a prior attempt to quit waterpipe smoking for more than 7 days compared to those with no previous attempt (OR: 6.60 CI: 1.32–32.96), and not smoking waterpipe when seriously ill compared to those who smoke even when seriously ill (OR: 6.50 CI: 1.40–30.09). Predictors associated with a lower intention to quit waterpipe smoking are increasing age (OR: 0.93 CI: 0.87–0.99), medium cultural acceptability of using waterpipe among family members compared to no-cultural acceptability among family (OR: 0.43 CI: 0.18–0.99), high cultural acceptability of using waterpipe among friends compared to no-cultural acceptability among friends (OR: 0.13 CI: 1.09–10.64), duration of smoking sessions between 1 hour (OR: 0.27 CI: 0.09–0.77) and 2 hours (OR: 0.04 CI: 0.00–0.93) compared to those who smoke for less than 30 minutes, and perception of waterpipe's harm as less than cigarettes (OR: 0.37 CI: 0.17–0.80) compared to those who perceive it as more harmful than cigarettes (Table 5).

## 4. Discussion

In our study sample of 340 Arab American waterpipe smokers, only 27.43% (*n* = 93) reported having an intention to quit, a percentage similar to that reported in a Syrian study [29].

The intention to quit waterpipe smoking in this study sample was significantly lower with increasing age. The finding contrasts previous studies on waterpipe smoking that reported no significant association between age and intention to quit [13, 29, 30]. A potential explanation to our finding could be that older participants in our sample could have spent a longer number of years using waterpipe and thus may have a higher addiction level. To confirm this potential assumption, we calculated the duration of waterpipe smoking by subtracting the age of initiating waterpipe smoking from the current age of the participants and we ran a bivariate regression between age and duration of waterpipe smoking (in years). The bivariate regression results indicated a significantly higher duration of waterpipe smoking in older participants for the study sample.

While no significant association between cigarette smoking in the last 30 days and an intention to quit waterpipe smoking was found in this study, a positive association between smoking cigars, cigarillos, or little cigars' in the last 30 days and the intention to quit waterpipe smoking was detected. The predominant intermittent use pattern of both cigar smoking and waterpipe smoking [31, 32] and the beliefs that they are less addictive and easier to quit compared to cigarette smoking [30, 32] might contribute to the positive association between being a cigar smoker and the intention to quit waterpipe smoking.

A previous attempt to quit has been reported as a significant predictor of an intention to quit tobacco smoking [33, 34]. This study also documents a higher intention to quit among those who had a previous attempt to stop waterpipe smoking for more than 7 days. Encouraging waterpipe smokers to try stopping, even once, could improve their chance of actually quitting in the future.

Findings of this research are also consistent with previous studies on waterpipe smoking that reported a lower intention to quit among smokers who used the waterpipe for more than 60 minutes per session [13]. In addition, waterpipe smokers who continue to smoke while ill were found to be less likely to have an intention to quit. This is potentially related to the association between longer duration of smoking or smoking even when seriously ill and higher nicotine exposure and dependence [13, 35], which has been shown to lower quit attempts among cigarette smokers [33, 34].

This study showed a higher intention to quit waterpipe smoking among those who perceived waterpipe use as more harmful than cigarettes compared to those who did not. This finding is consistent with prior research [13]. In addition, lesser harm perception compared to cigarettes has also been identified as a predictor of use for waterpipe smokers in a number of studies in the US and abroad [16, 36].

As a factor influential in both initiation and continuation of waterpipe smoking, educating and informing users of potential harms and negative health consequences should be part of any intervention aiming at increasing the intention to quit waterpipe smoking as an initial step to quitting.

Finally, the results of this study demonstrated the significant effect of the social norms on the smoker's intention to quit waterpipe smoking as previously described in literature [29, 30]. Arab Americans whose friends and family members have a medium or high social acceptability of waterpipe smoking had a lesser intention to quit. Waterpipe smoking has been shown to be part of a social phenomenon as it is frequently carried out in groups and gatherings thus making the habit more appealing [27, 37, 38]. This social aspect has to be taken into consideration when designing any intervention to promote quitting. Including friends and family members in the programs and interventions that aim to increase the smokers' awareness about the negative aspects of smoking could be beneficial.

## 5. Limitations and Future Research

The study suffers the limitations of the cross-sectional design as we are unable to draw causal associations with such a design. As the study was conducted using a convenience sample of Arab Americans in Houston, Texas area only, the generalizability of the finding may be limited to this geographic area and not to all Arab Americans. The replication of this study in different settings and geographic locations would provide better generalizability of the results.

Our study did not address some important factors related to waterpipe smoking, such as reasons for starting smoking, history of any health complications due to waterpipe smoking, motivations to quit waterpipe smoking, type of product used in waterpipe smoking (herbal versus tobacco), difference between herbal products' smokers and tobacco products' smokers intention to quit, and the intention to quit other tobacco products if smoking any. Future research projects that include assessing these factors among Arab American waterpipe smokers are needed.

In addition, we relied on each subject's self-reported data, which might contain some potential sources of bias, such as selective memory (to remember or not remember experiences or events that occurred at some point in the past) or social desirability bias as a result of the tendency of smokers to base their answers on what they think is theoretically right not what they usually do.

## 6. Conclusion

Findings of this study enriched current knowledge about the intention to quit waterpipe smoking with two important additions. First, history of cigar use, a prior attempt to quit, and not smoking waterpipe when seriously ill were found to significantly increase the intention to quit waterpipe smoking. Second, significantly lower intentions to quit waterpipe smoking were indicated with increasing age, medium cultural acceptability of using waterpipe among family members, high cultural acceptability of using waterpipe among friends, longer duration of smoking sessions, and perceiving waterpipe smoking as less harmful than cigarettes.

Our findings demonstrate low levels of an intention to quit waterpipe smoking among Arab Americans in the Houston, Texas area. Public health educational programs and interventions that target Arab Americans in general, and specifically older adults, those who smoke waterpipe for more than 60 minutes, those whose family and friends approve of waterpipe smoking, and those with no former attempts to quit, may be necessary to increase the intention to quit waterpipe smoking as the first step of quitting.

## Figures and Tables

**Table 1:** Frequency of intention to quit waterpipe smoking by sample characteristics.

Characteristics	Has intention to quit in the next 12 months		
	(No) frequency (%)	(Yes) frequency (%)	*P* value
Total	246 (72.57%)	93 (27.43%)	
Gender			0.2482
Male	161 (70.61)	67 (29.39)	
Female	85 (76.58)	26 (23.42)	
Income level			0.2685
<$20,000	64 (68.09)	30 (31.91)	
$20,000–$35,000	38 (69.69)	17 (30.91)	
$35,000–$50,000	46 (73.02)	17 (26.98)	
$50,000–$100,000	53 (77.94)	15 (22.06)	
>$100,000	37 (84.09)	7 (15.91)	
Marital status			0.6512
Single	112 (71.34)	45 (28.66)	
Married	124 (73.81)	44 (26.19)	
Divorced	8 (80.00)	2 (20.00)	
Widowed	3 (100.00)	0 (0.00)	
Education level			0.7195
<High school degree	7 (63.64)	4 (36.36)	
High school degree	23 (67.65)	11 (32.35)	
College or university degree	146 (72.64)	55 (27.36)	
Graduate degree (MS or PhD)	69 (75.82)	22 (24.18)	
Do you usually share the waterpipe with others?			0.0675
Yes	149 (69.30)	66 (30.70)	
No	86 (78.90)	23 (21.10)	
Do you own a waterpipe?			0.2426
Yes	168 (74.67)	57 (25.33)	
No	79 (68.70)	36 (31.30)	
Is the tobacco used in waterpipe flavored?			0.1312
Yes	210 (73.68)	75 (26.32)	
No	18 (58.06)	13 (41.94)	
Sometimes	13 (81.25)	3 (18.75)	
Smoked cigarettes in the last 30 days			0.2993
Yes	115 (70.55)	48 (29.45)	
No	130 (75.58)	42 (24.42)	
Smoked cigars, cigarillos, or little cigars in the last 30 days			0.2065
Yes	57 (55.34)	46 (44.66)	
No	145 (62.50)	87 (37.50)	

**Table 2:** Frequency intention to quit waterpipe smoking with perception of risk.

Characteristics	Has intention to quit in the next 12 months		
	(No) frequency (%)	(Yes) frequency (%)	*P* value
Do you believe smoking a waterpipe is harmful to your health?			0.3576
Yes	202 (72.40)	77 (27.60)	
No	34 (79.07)	9 (20.93)	
Compared to a regular cigarette, how harmful do you think waterpipe smoking is?			0.0022^*^
More harmful than cigarettes	87 (65.55)	41 (34.45)	
As harmful as cigarettes	74 (67.89)	35 (32.11)	
Less harmful than cigarettes	90 (84.91)	16 (15.09)	

^*^Significant at *P* < 0.05.

**Table 3:** Frequency of intention to quit waterpipe smoking with perceived social acceptability.

Characteristics	Has intention to quit in the next 12 months		
	(No) frequency (%)	(Yes) frequency (%)	*P* value
The social acceptability of using a waterpipe among your peers?			0.2031
None	25 (69.44)	11 (30.56)	
Low	55 (73.33)	20 (26.67)	
Medium	79 (67.52)	38 (32.48)	
High	82 (79.61)	21 (20.39)	
The cultural acceptability of using a waterpipe among your family members?			0.0028^*^
None	28 (57.14)	21 (42.86)	
Low	65 (65.66)	34 (34.34)	
Medium	99 (81.15)	23 (18.85)	
High	49 (79.03)	13 (20.97)	
The cultural acceptability of using a waterpipe among friends of your ethnicity?			0.0158^*^
None	20 (62.50)	12 (37.50)	
Low	24 (58.54)	17 (41.46)	
Medium	89 (71.20)	36 (28.80)	
High	109 (80.74)	26 (19.26)	
How often do you attend Middle Eastern gatherings where waterpipe are served?			0.2615
Daily	34 (62.96)	20 (37.04)	
Weekly	119 (76.77)	36 (23.23)	
Monthly	59 (71.08)	24 (28.92)	
Yearly	27 (71.05)	11 (28.95)	
How cool do your peers look when they use the waterpipe?			0.1377
Not at all	81 (71.68)	32 (28.32)	
Cool	119 (69.59)	52 (30.41)	
Very cool	38 (84.44)	7 (15.56)	

^*^Significant at *P* < 0.05.

**Table 4:** Frequency of intention to quit waterpipe smoking with different waterpipe-related practices and beliefs.

Characteristics	Has intention to quit		
	(No) frequency (%)	(Yes) frequency (%)	*P* value
The likelihood of getting addicted when using a waterpipe socially			0.6314
None	49 (75.38)	16 (24.62)	
Low	90 (75.00)	30 (25.00)	
Medium	67 (68.37)	31 (31.63)	
High	38 (76.00)	12 (24.00)	
The likelihood of getting addicted when using a waterpipe by oneself			0.2538
None	57 (76.00)	18 (24.00)	
Low	94 (78.33)	26 (21.67)	
Medium	62 (67.39)	30 (32.61)	
High	30 (68.18)	14 (31.82)	
A governmental agency should evaluate the safety of the waterpipe before selling it			0.3104
Yes	151 (70.89)	29 (23.97)	
No	92 (76.03)	62 (29.11)	
How often you smoke tobacco using a waterpipe?			0.0912
At least once a year but not monthly	32 (60.38)	21 (39.62)	
At least once a month but not weekly	55 (68.75)	25 (31.25)	
At least once a week but not daily	96 (75.59)	31 (24.41)	
At least once a day, or most days each month	59 (78.67)	16 (21.33)	
How long did a typical “waterpipe session” last?			0.0004^*^
0–30 minutes	46 (57.50)	34 (42.50)	
31–60 minutes	84 (70.59)	35 (29.41)	
61–90 minutes	73 (85.88)	12 (14.12)	
91–120 minutes	24 (82.76)	5 (17.24)	
120+ minutes	18 (85.71)	3 (14.29)	
Do you consider yourself “hooked” on a waterpipe?			0.7737
Yes	80 (74.77)	27 (25.23)	
No	159 (73.27)	58 (26.73)	
Number of times you could stop waterpipe for more than 7 days			0.0137^*^
None	44 (81.48)	10 (18.52)	
Once	14 (48.28)	15 (51.72)	
Several times	83 (72.81)	31 (27.19)	
It always happens	98 (73.13)	36 (26.87)	
Percentage of income would you spend for waterpipe smoking			0.4706
1% or less of your monthly income	131 (70.05)	56 (29.95)	
2%–10% of your monthly income	79 (78.22)	22 (21.78)	
11%–50% of your monthly income	19 (67.86)	9 (32.14)	
More than 50% of your monthly income	11 (73.33)	4 (26.67)	
Number of days you could spend without waterpipe?			0.7587
One day or less	29 (76.32)	9 (23.68)	
2-3 days	40 (67.80)	19 (32.20)	
4–7 days	28 (70.00)	12 (30.00)	
More than 7 days	145 (73.60)	52 (26.40)	
Number of waterpipes you usually smoke per week			0.4480
<1 waterpipe/week	92 (69.70)	40 (30.30)	
1-2 waterpipes/week	59 (71.08)	24 (28.92)	
3–6 waterpipes/week	59 (78.67)	16 (21.33)	
7 or more waterpipes/week	29 (78.38)	8 (21.62)	
Do you smoke waterpipe to relax your nerves?			0.1978
Yes, absolutely	43 (84.31)	8 (15.69)	
Yes, probably	71 (68.93)	32 (31.07)	
Yes, maybe	59 (69.41)	26 (30.59)	
No	71 (73.20)	26 (26.80)	
Do you smoke waterpipe to improve your morale?			0.2342
Yes, absolutely	12 (75.00)	4 (25.00)	
Yes, probably	34 (68.00)	16 (32.00)	
Yes, maybe	40 (64.52)	22 (35.48)	
No	157 (76.59)	48 (23.41)	
Do you smoke waterpipe when you are seriously ill?			0.9232
Yes, absolutely	10 (71.43)	4 (28.57)	
Yes, probably	23 (67.65)	11 (32.35)	
Yes, maybe	28 (73.68)	10 (26.32)	
No	182 (73.09)	67 (26.91)	
Do you smoke waterpipe alone?			0.3140
Yes, absolutely	28 (84.85)	5 (15.15)	
Yes, probably	29 (65.91)	15 (34.09)	
Yes, maybe	77 (71.96)	30 (28.04)	
No	102 (71.33)	41 (28.67)	
Are you ready not to eat in exchange for a waterpipe?			0.3187
Yes, absolutely	17 (62.96)	10 (37.04)	
Yes, probably	25 (64.10)	14 (35.90)	
Yes, maybe	27 (77.14)	8 (22.86)	
No	165 (74.66)	56 (25.34)	
Do you smoke waterpipe for pleasure?			0.0189^*^
Yes, absolutely	104 (80.00)	26 (20.00)	
Yes, probably	54 (69.23)	24 (30.77)	
Yes, maybe	53 (73.61)	19 (26.39)	
No	28 (57.14)	21 (42.86)	
Do you smoke waterpipe to please others?			0.0264^*^
Yes, absolutely	11 (64.71)	6 (35.29)	
Yes, probably	10 (47.62)	11 (52.38)	
Yes, maybe	17 (65.38)	9 (34.62)	
No	204 (75.84)	65 (24.16)	

^*^Significant at *P* < 0.05.

**Table 5:** Results of bivariate and multiple logistic regressions to predict intention to quit waterpipe smoking.

Characteristics	Unadjusted OR (CI 95%) for variables with *P* < 0.2	Adjusted OR (CI 95%)^a^ for variables with *P* < 0.05
Age	0.96 (0.93–0.99)^*^	0.93 (0.87–0.99)^*^
Income level		
<$20,000	Reference	
$20,000–$35,000	0.95 (0.46–1.95)	
$35,000–$50,000	0.78 (0.38–1.59)	
$50,000–$100,000	0.60 (0.29–1.23)	
>$100,000	0.40 (0.16–1.01)	
Smoked cigars, cigarillos, or little cigars in the last 30 days		
Yes	2.31 (1.39–3.82)^**^	4.38 (1.86–10.31)^***^
No	Reference	Reference
The social acceptability of using a waterpipe among your peers?		
None	Reference	
Low	0.82 (0.34–1.98)	
Medium	1.09 (0.48–2.45)	
High	0.58 (0.24–1.37)	
The cultural acceptability of using a waterpipe among your family members?		
None	Reference	Reference
Low	0.69 (0.34–1.40)	2.24 (0.51–9.74)
Medium	0.31 (0.15–0.64)^*^	0.43 (0.18–0.99)^*^
High	0.35 (0.15–0.81)^*^	0.33 (0.05–1.98)
The cultural acceptability of using a waterpipe among friends of your ethnicity?		
None	Reference	Reference
Low	1.18 (0.45–3.04)	0.70 (0.11–4.16)
Medium	0.67 (0.29–1.52)	0.20 (0.03–1.03)
High	0.39 (0.17–0.91)^**^	0.13 (1.09–10.64)^*^
How often do you attend Middle Eastern gatherings where waterpipe are served?		
Daily	Reference	
Weekly	0.51 (0.26–1.00)	
Monthly	0.69 (0.33–1.43)	
Yearly	0.69 (0.28–1.69)	
How cool do your peers look when they use the waterpipe?		
Not at all	Reference	
Cool	1.10 (0.65–1.86)	
Very cool	0.46 (0.18–1.15)	
How often you smoke tobacco using a waterpipe?		
At least once a year but not monthly	Reference	
At least once a month but not weekly	0.69 (0.33–1.43)	
At least once a week but not daily	0.49 (0.24–0.97)^*^	
At least once a day, or most days each month	0.41 (0.18–0.90)^*^	
How long did a typical “waterpipe session” last?		
0–30 minutes	Reference	Reference
31–60 minutes	0.56 (0.31–1.02)	0.41 (0.16–1.06)
61–90 minutes	0.22 (0.10–0.47)^*^	0.270 (0.09–0.77)^*^
91–120 minutes	0.28 (0.09–0.81)^*^	0.044 (0.00–0.93)^*^
120+ minutes	0.22 (0.06–0.82)^*^	0.234 (0.02–2.80)
Do you usually share the waterpipe with others?		
Yes	1.65 (0.96–2.85)	
No	Reference	
Number of times you could stop waterpipe for more than 7 days		
None	Reference	Reference
Once	4.71 (1.73–12.82)^**^	6.60 (1.32–32.96)^*^
Several times	1.64 (0.73–3.66)	2.36 (0.55–10.07)
It always happens	1.61 (0.73–3.54)	1.34 (0.33–5.35)
Do you smoke waterpipe when you are seriously ill?		
Yes, absolutely	Reference	Reference
Yes, probably	1.19 (0.30–4.67)	0.06 (0.01–1.42)
Yes, maybe	0.89 (0.22–3.50)	0.15 (0.01–1.36)
No	0.51 (0.17–1.52)	6.50 (1.40–30.09)^**^
Do you smoke waterpipe for pleasure?		
Yes, absolutely	Reference	
Yes, probably	1.77 (0.93–3.38)	
Yes, maybe	1.43 (0.72–2.82)	
No	3.00 (1.47–6.10)^*^	
Do you smoke waterpipe to please others?		
Yes, absolutely	Reference	
Yes, probably	2.01 (0.54–7.49)	
Yes, maybe	0.97 (0.26–3.49)	
No	0.58 (0.20–1.64)^**^	
The likelihood of getting addicted when using a waterpipe socially		
None	Reference	
Low	1.02 (0.50–2.05)	
Medium	1.41 (0.69–2.87)	
High	0.96 (0.40–2.28)	
The likelihood of getting addicted when using a waterpipe by oneself		
None	Reference	
Low	0.87 (0.44–1.73)	
Medium	1.53 (0.77–3.04)	
High	1.47 (0.64–3.37)	
Do you believe smoking a waterpipe is harmful to your health?		
Yes	1.44 (0.66–3.14)	
No	Reference	
Compared to a regular cigarette, how harmful do you think waterpipe smoking is?		
More harmful than cigarettes	Reference	Reference
As harmful as cigarettes	0.90 (0.51–1.56)	1.16 (0.61–2.22)
Less harmful than cigarettes	0.33 (0.17–0.64)^***^	0.37 (0.17–0.80)^**^

^a^Adjusted to all variables with unadjusted odds ratio with *P* < 0.2.

^*^ *P* < 0.05, ^**^ *P* < 0.01, and ^***^ *P* < 0.001.
